# Module M1 of Zebrafish Neuroglobin Acts as a Structural and Functional Protein Building Block for a Cell-Membrane-Penetrating Activity

**DOI:** 10.1371/journal.pone.0016808

**Published:** 2011-02-03

**Authors:** Seiji Watanabe, Keisuke Wakasugi

**Affiliations:** 1 Department of Life Sciences, Graduate School of Arts and Sciences, The University of Tokyo, Tokyo, Japan; 2 Precursory Research for Embryonic Science and Technology (PRESTO), Japan Science and Technology Agency (JST), Saitama, Japan; University Paris Sud, France

## Abstract

Neuroglobin (Ngb) is a recently discovered vertebrate globin that is expressed in the brain and can reversibly bind oxygen. Mammalian Ngb is involved in neuroprotection during oxidative stress that occurs, for example, during ischemia and reperfusion. Recently, we found that zebrafish, but not human, Ngb can translocate into cells. Moreover, we demonstrated that a chimeric ZHHH Ngb protein, in which the module M1 of human Ngb is replaced by the corresponding region of zebrafish Ngb, can penetrate cell membranes and protect cells against oxidative stress-induced cell death, suggesting that module M1 of zebrafish Ngb is important for protein transduction. Furthermore, we recently showed that Lys7, Lys9, Lys21, and Lys23 in module M1 of zebrafish Ngb are crucial for protein transduction activity. In the present study, we have investigated whether module M1 of zebrafish Ngb can be used as a building block to create novel cell-membrane-penetrating folded proteins. First, we engineered a chimeric myoglobin (Mb), in which module M1 of zebrafish Ngb was fused to the N-terminus of full-length human Mb, and investigated its functional and structural properties. Our results showed that this chimeric Mb protein is stable and forms almost the same heme environment and α-helical structure as human wild-type Mb. In addition, we demonstrated that chimeric Mb has a cell-membrane-penetrating activity similar to zebrafish Ngb. Moreover, we found that glycosaminoglycan is crucial for the cell-membrane-penetrating activity of chimeric Mb as well as that of zebrafish Ngb. These results enable us to conclude that such module substitutions will facilitate the design and production of novel functional proteins.

## Introduction

Globins are iron porphyrin complex (heme)-containing proteins that bind reversibly to oxygen (O_2_) and, as such, play an important role in respiratory function. Neuroglobin (Ngb) is a recently discovered globin found in the vertebrate brain that has a high affinity for oxygen [1]–[3]. Ngb is widely expressed in the cerebral cortex, hippocampus, thalamus, hypothalamus, cerebellum, and retina [1], [4]–[6]. It was recently suggested that mammalian Ngb might be involved in the neuronal response to hypoxia and ischemia [7]–[12]. Mammalian Ngb expression has been reported to increase in response to neuronal hypoxia *in vitro* and to ischemia *in vivo* [7], [8], [12]. Neuronal survival after hypoxia or oxidative stress was reduced by inhibiting Ngb expression with an antisense oligodeoxynucleotide and was enhanced by Ngb overexpression, supporting the notion that mammalian Ngb protects neurons from hypoxic-ischemic insults [7], [9], [11]. Mammalian Ngb has been reported to protect the brain from experimentally induced stroke *in vivo* [8], [10]. We previously found that human Ngb binds exclusively to the GDP-bound form of the α-subunit of heterotrimeric G protein (Gα_i_) and acts as a guanine nucleotide dissociation inhibitor (GDI) by inhibiting the rate of exchange of GDP for GTP on Gα_i_ [13]–[15]. Recently, we used a protein delivery reagent, Chariot [16], to investigate whether the GDI activity of human Ngb plays an important role in its neuroprotective activity under oxidative stress conditions. As a result, we demonstrated that the GDI activity of human Ngb is tightly correlated with its neuroprotective activity [17].

Although Ngb was originally identified in mammalian species, it is also present in non-mammalian vertebrates, including the zebrafish [18], [19]. Mammalian and fish Ngb proteins share about 50% amino acid sequence identity. Fish Ngb has oxygen-binding kinetics similar to mammalian Ngb [19]. We previously showed that zebrafish Ngb lacks GDI activity and that zebrafish Ngb cannot rescue cell death significantly under oxidative stress conditions as compared with human Ngb [17], [20], [21].

Unlike the genes of prokaryotes whose coding sequences are continuous, the coding sequences of eukaryotic genes have been found to be present in blocks, ‘exons’, separated by intervening noncoding sequences, ‘introns’. Gilbert and Blake hypothesized that exons encode functional and structural units, and that new functional proteins have evolved through the selection of various combinations of these units that are produced by unequal crossing-over on introns—a process that is termed ‘exon shuffling’ [22], [23]. Using a diagonal plot of all of the distances between the α-carbon atoms, Gō demonstrated that there is a correlation between protein structure and exon pattern, and found that the ‘modules’, which could be called compact structural units, correspond to the exons [24]. The correspondence of the ‘modular’ boundaries with the positions of introns in globins, lysozyme,cytochrome c, and other proteins suggests that exons may have behaved as evolutionary units to produce new proteins by combining various exons through the mechanism of exon shuffling [24]–[26]. The genes of both human and zebrafish Ngb are made up of four exons interrupted by three introns, and exons 1, 2, 3, and 4 encode compact protein structural ‘modules’, termed M1, M2, M3, and M4, respectively [18]–[20], [24].

Previously, we engineered a chimeric ZHHH Ngb protein, in which module M1 of human Ngb was replaced by that of zebrafish Ngb, and showed that this chimeric ZHHH Ngb forms almost the same structure as human Ngb and acts as a GDI for Gα_i_ in a manner similar to human Ngb [20]. We showed that protein transduction of chimeric ZHHH Ngb, but not zebrafish Ngb with a protein delivery reagent, Chariot, rescued PC12 cell death caused by hypoxia/reoxygenation [17]. Moreover, we discovered that chimeric ZHHH Ngb protects PC12 cells against oxidative stress-induced cell death even in the absence of Chariot [21]. By using fluorescein isothiocyanate (FITC)-labeled Ngb proteins, we demonstrated that both zebrafish and chimeric ZHHH Ngb could penetrate cell membranes in the absence of Chariot [21]. This finding suggested that module M1 of zebrafish Ngb is essential for protein transduction into cells, because both the zebrafish and chimeric ZHHH Ngb proteins share this module. Furthermore, we recently showed that residues Lys7, Lys9, Lys21, and Lys23 in module M1 of zebrafish Ngb are crucial for protein transduction activity [27].

**Figure 1:**
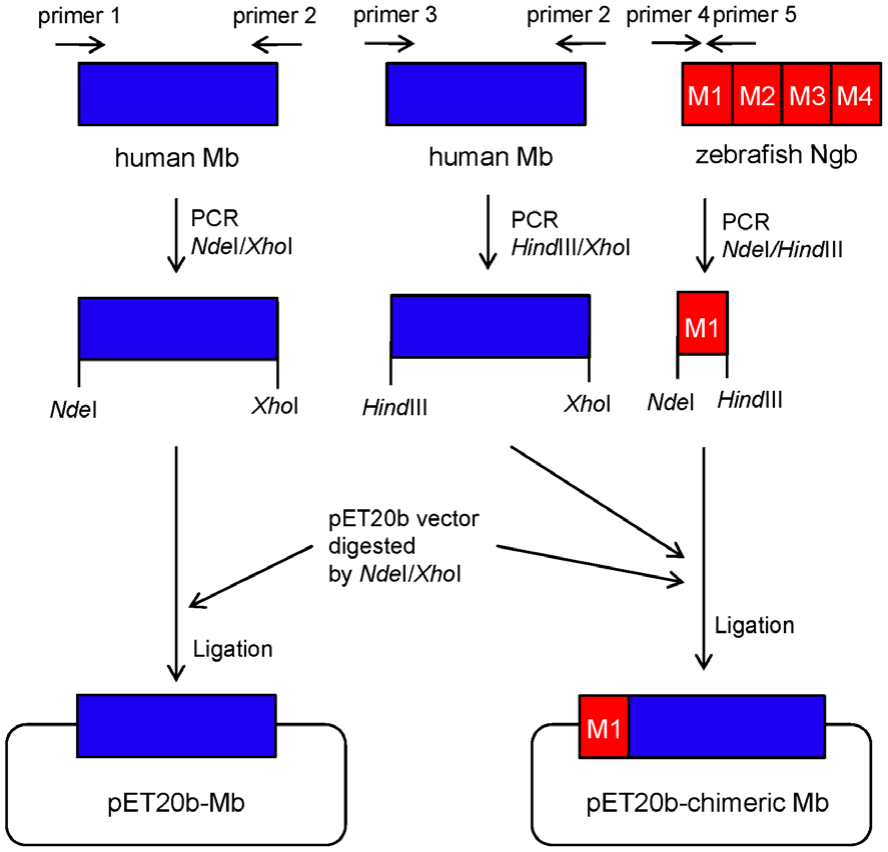
Schematic representation of preparation of expression constructs encoding chimeric Mb and wild-type Mb. The primers used for PCR were as follows. Primer 1: 5′- GGAATTCCATATGCTCAGCGACGGGGAATGGCAGTTGGTGCTGAACG-3′; primer 2: 5′- GCCGCTCGAGGCCCTGGAAGCCCAGCTCCTTGTAGTTGGAGGCCATGTCC-3′; primer 3: 5′- CGGCCAAGCTTGGGCTCAGCGACGGGGAATGGCAGTTGGTGCTGAACG-3′; primer 4: 5′- GGAATTCCATATGGAGAAGCTGTCTGAAAAAGATAAGGGTCTCATCCGGGACAGCTGGG-3′; primer 5: 5′- CGGCCAAGCTTCGTGAACAAAACGATTCCATGTGGCACCTTGTTCTTCCCCAGACTCTC-3′.

The objective of this study was to investigate whether module M1 of zebrafish Ngb can be used as a building block to create novel cell-membrane-penetrating, folded proteins. To that end, we engineered a myoglobin (Mb) variant, hereafter termed ‘chimeric Mb’, in which the M1 module of zebrafish Ngb was fused to the N-terminus of full-length human Mb, as shown in Figure 1, and characterized its structural and functional properties. Our results showed that chimeric Mb is stable and forms almost the same heme environment and secondary structure as the human Mb protein. In addition, we demonstrated that chimeric Mb has a cell-membrane-penetrating activity similar to zebrafish Ngb. Moreover, we tried to identify negatively-charged cell surface molecules that can interact with the Lys residues of either zebrafish Ngb or chimeric Mb for protein transduction. We show that glycosaminoglycan is crucial for the cell-membrane-penetrating activity of chimeric Mb, as well as zebrafish Ngb. These results enable us to conclude that such module substitutions represent a potent strategy to design and produce stable functional proteins.

## Results and Discussion

### Purification and association properties of chimeric Mb

**Figure 2:**
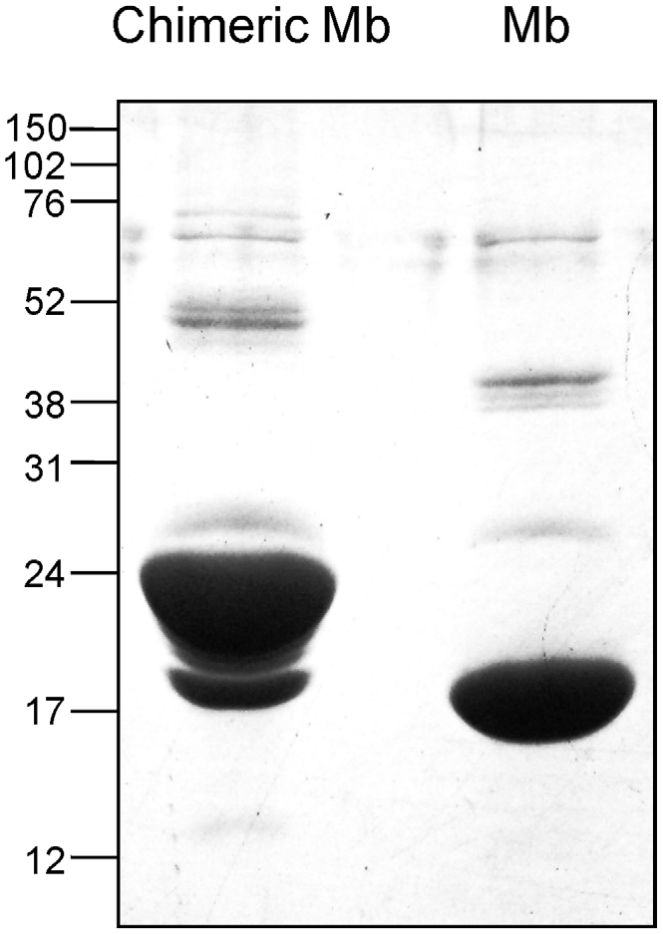
SDS-PAGE analysis of chimeric and human wild-type Mb. The samples were analyzed on a 15.0% SDS-polyacrylamide gel and stained with Coomassie Blue. Molecular size markers are shown at the left (in kilodaltons).

**Figure 3:**
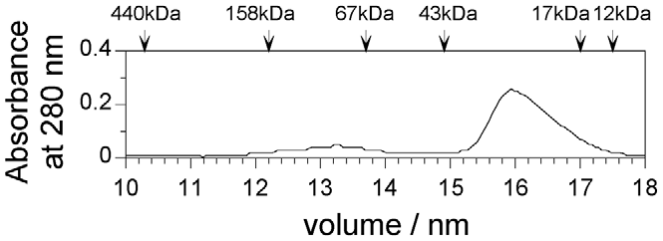
Gel filtration chromatography of chimeric Mb on a Superdex 200 column of the FPLC system. Chimeric Mb was loaded onto the column equilibrated with 20 mM Tris-HCl and 150 mM NaCl, pH 8.0, at 4°C. The optical density profiles were monitored at 280 nm. The elution volumes of ferritin (440 kDa), aldolase (158 kDa), bovine serum albumin (67 kDa), ovalbumin (43 kDa), horse heart myoglobin (17 kDa), and bovine cytochrome c (12 kDa) were 10.3, 12.2, 13.7, 14.9, 17.0, and 17.5 ml, respectively, and these data were used for column calibration.

The chimeric and human wild-type Mb proteins, including a COOH-terminal tag of six histidine residues (six-His tag), were expressed in *E. coli* and purified, and their molecular size (expected molecular size: 22 kDa (chimeric Mb), 18 kDa (wild-type Mb)) and purity were confirmed by 15.0% SDS–polyacrylamide gel electrophoresis (SDS-PAGE) (Figure 2). The association properties of chimeric Mb were examined by gel filtration over a calibrated FPLC Superdex 200 column. As shown in Figure 3, chimeric Mb eluted mainly at a fraction corresponding to the monomer (22 kDa).

### Electronic absorption spectra of chimeric Mb

**Figure 4:**
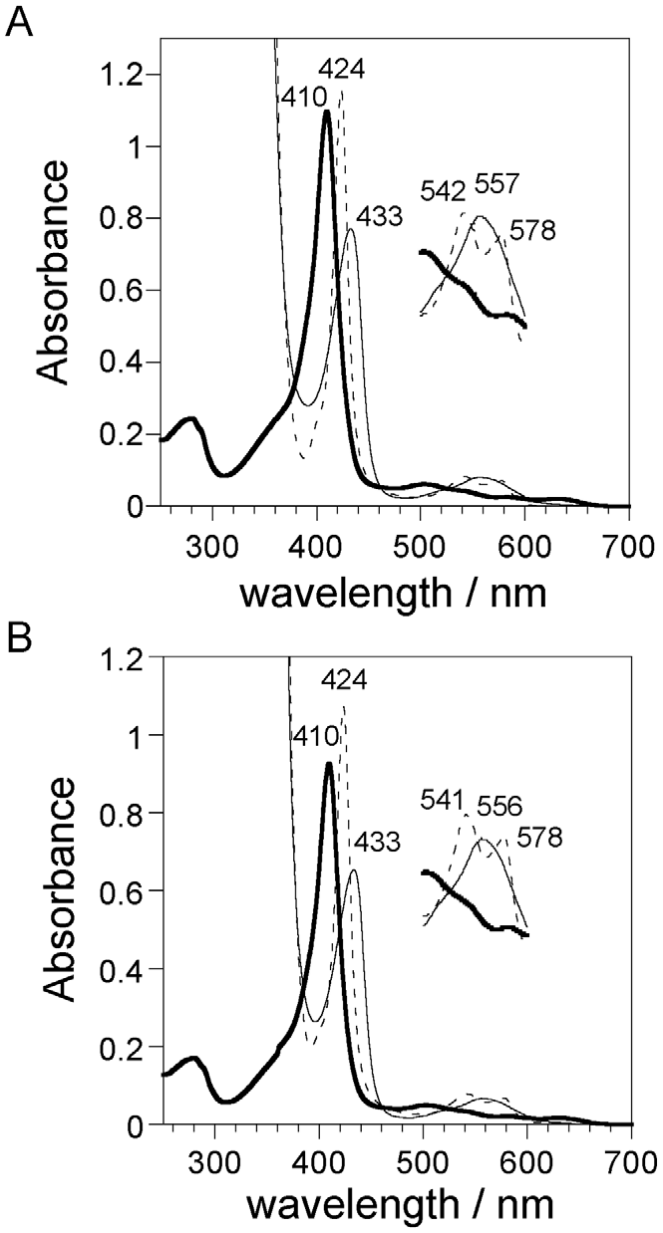
Electronic absorption spectra of the ferric (bold line), ferrous deoxy (fine line), and ferrous-CO (dotted line) forms of chimeric (A) and human wild-type Mb (B). The Q bands from 500 to 600 nm are enlarged by a factor of 5 on the perpendicular axis. The spectra were recorded in PBS (pH 7.4) at ambient temperature (∼20°C).

Initially, we determined the effects of the M1 module on the electronic state of the heme by measuring the absorption spectra of the chimeric Mb protein. As shown in Figure 4, the UV-visible spectra of the ferric, ferrous deoxy, and ferrous carbon monoxide-bound (ferrous-CO) forms of chimeric Mb were nearly identical to those of human wild-type Mb. In addition, the wavelengths and extinction coefficients at the absorption maxima for chimeric Mb were almost the same as those for the wild-type protein, demonstrating that the heme environment in chimeric Mb was almost the same as that in wild-type Mb.

### CD spectra of chimeric Mb

**Figure 5:**
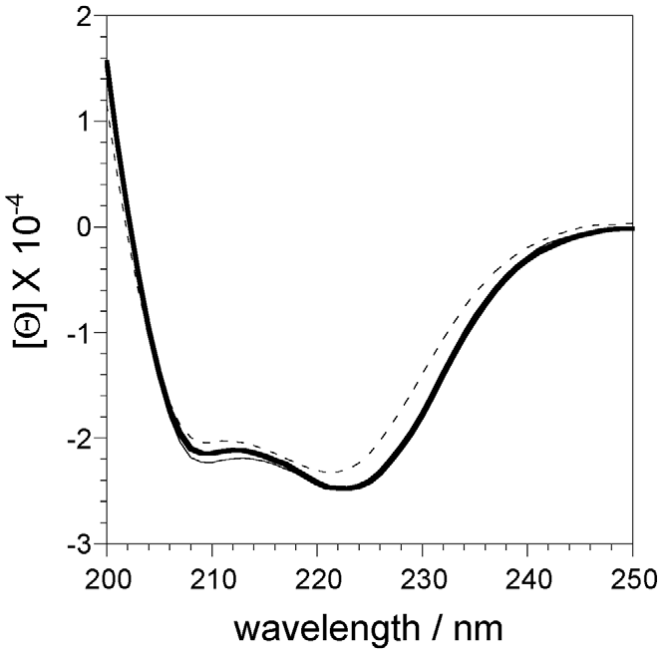
Circular dichroism (CD) spectra in the far UV region of the ferric form of chimeric Mb (bold line), human wild-type Mb (fine line), and zebrafish wild-type Ngb (dotted line). The concentration of each protein was approximately 5 µM on the basis of heme content. The spectra were recorded in 50 mM sodium phosphate buffer (pH 7.4) at 20°C.

Next, to examine the effects of module M1 attachment on the secondary structure of globin, we measured the far UV CD spectra of the ferric forms of chimeric Mb, human wild-type Mb and zebrafish wild-type Ngb. As shown in Figure 5, human wild-type Mb and zebrafish wild-type Ngb exhibited two negative broad peaks around 222 and 208 nm, which are characteristic of an α-helical structure. These negative peaks were also observed in chimeric Mb (Figure 5). The α-helical content of the chimeric Mb protein was estimated to be 74%, which is almost the same as those of human wild-type Mb (74%) and zebrafish Ngb (69%). These results showed that the secondary protein structure was insensitive to attachment of the module M1.

### Denaturation properties of chimeric Mb

**Figure 6:**
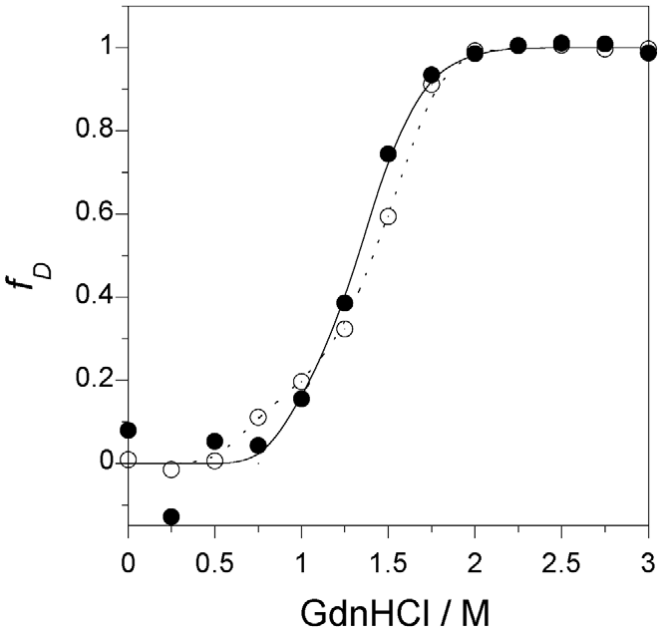
GdnHCl denaturation curves of wild-type and chimeric Mb. Denaturation curves were measured for human (open circle), and chimeric (closed circle) Mb. Molecular ellipticities at 222 nm in the native and completely denatured states were normalized to 0 and 1, respectively. The concentration of each protein was 5 µM on the basis of heme content.

Alterations in equilibrium stability caused by the attachment of M1 were quantified in a GdnHCl-induced denaturation experiment. The GdnHCl denaturation process was followed by monitoring ellipticity at 222 nm, which reflects structural changes in the whole protein. As shown in Figure 6, the ferric forms of wild-type and chimeric Mb showed cooperative transition curves. The transition curve for GdnHCl denaturation of chimeric Mb was similar to that for human wild-type Mb, indicating that the globular structure of chimeric Mb was as stable as that of wild-type Mb.

### Cell-membrane-penetrating activity of chimeric Mb

**Figure 7:**
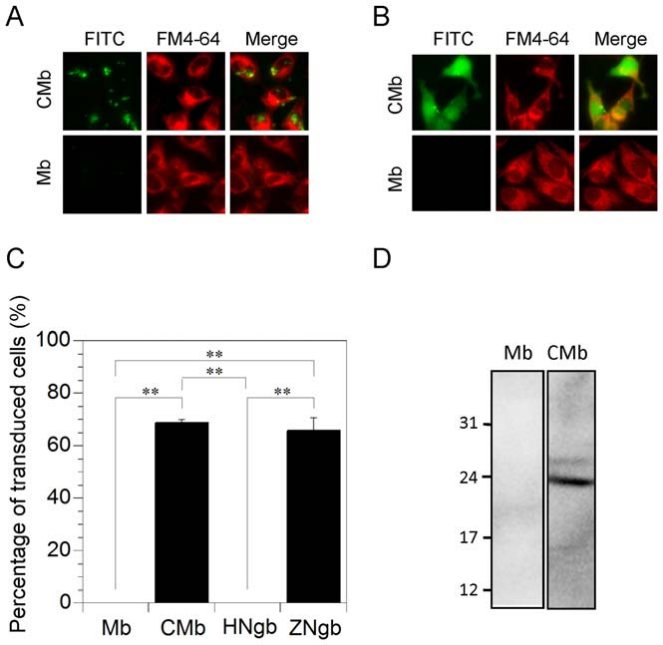
Transduction of human wild-type Mb or chimeric Mb (CMb) into HeLa cells. (A,B) Images of FITC-labeled Mb or CMb in HeLa cells. Each FITC-labeled (green) protein was applied at 1 µM to HeLa cells seeded on glass in the presence of FM4-64 (red), a fluorescent marker of endocytosis. The cells were then incubated for 6 h (A) or 24 h (B) under normoxic conditions. The living, unfixed cells were directly observed by fluorescence microscopy. (C) Percentages of transduced cells showing FITC signal after a 24-h incubation in the fluorescence microscopy assays, from a random selection of fields including at least one hundred cells in total. All data are expressed as means ± standard error of means (SEM) from three independent experiments. ** *P*<0.01, one-way ANOVA. (D) Western blot analyses of Mb and CMb, which were transduced into cells. Mb or CMb was applied at 5 µM to HeLa cells. The cells were then incubated for 24 h. Protein samples were analyzed on 18.0% SDS-polyacrylamide gels and by Western blot analysis using anti-His monoclonal antibody. Molecular size markers (in kilodaltons) are shown on the left.

We previously showed that wild-type zebrafish Ngb labeled with FITC could translocate into cells [21]. Here we evaluated the effects of module M1 on the translocation properties of wild-type Mb. The chimeric and wild-type Mb proteins were fluorescently labeled to assess their transduction efficiency in living cells. Figures 7A and 7B show that chimeric Mb had translocated through the cell membrane after 6 h of incubation. In contrast, wild-type Mb had not translocated into HeLa cells even after 24 h of incubation (Figure 7B). Figure 7C shows that the translocation efficacy of chimeric Mb is almost the same as that of zebrafish Ngb. Moreover, western blot analyses of lysates of transduced cells demonstrated that intact chimeric Mb was delivered to the cytosol (Figure 7D).

### Glycosaminoglycan is crucial for the cell-membrane-penetrating activity of zebrafish Ngb and chimeric Mb

BETA2/NeuroD and human immunodeficiency virus type 1 (HIV-1) TAT (transactivator of transcription) proteins permeate cells owing to the presence of arginine (Arg)- and lysine (Lys)-rich protein transduction domains [28]–[30]. The sequence of zebrafish Ngb module M1 shares several conserved Arg and Lys residues with other fish Ngb proteins [21]. Moreover, we recently performed site-directed mutagenesis of Arg and Lys residues within the M1 module to identify amino acid residues critical for protein transduction and showed that Lys7, Lys9, Lys21, and Lys23 in module M1 of zebrafish Ngb are essential for protein transduction activity [27]. Because it has been reported that negatively-charged cell surface membrane-associated proteoglycans are required for internalization of BETA2/neuroD protein or TAT peptide [31], [32], we first tried to identify negatively-charged cell surface molecules that can interact with the Lys residues of zebrafish Ngb for protein transduction.

**Figure 8:**
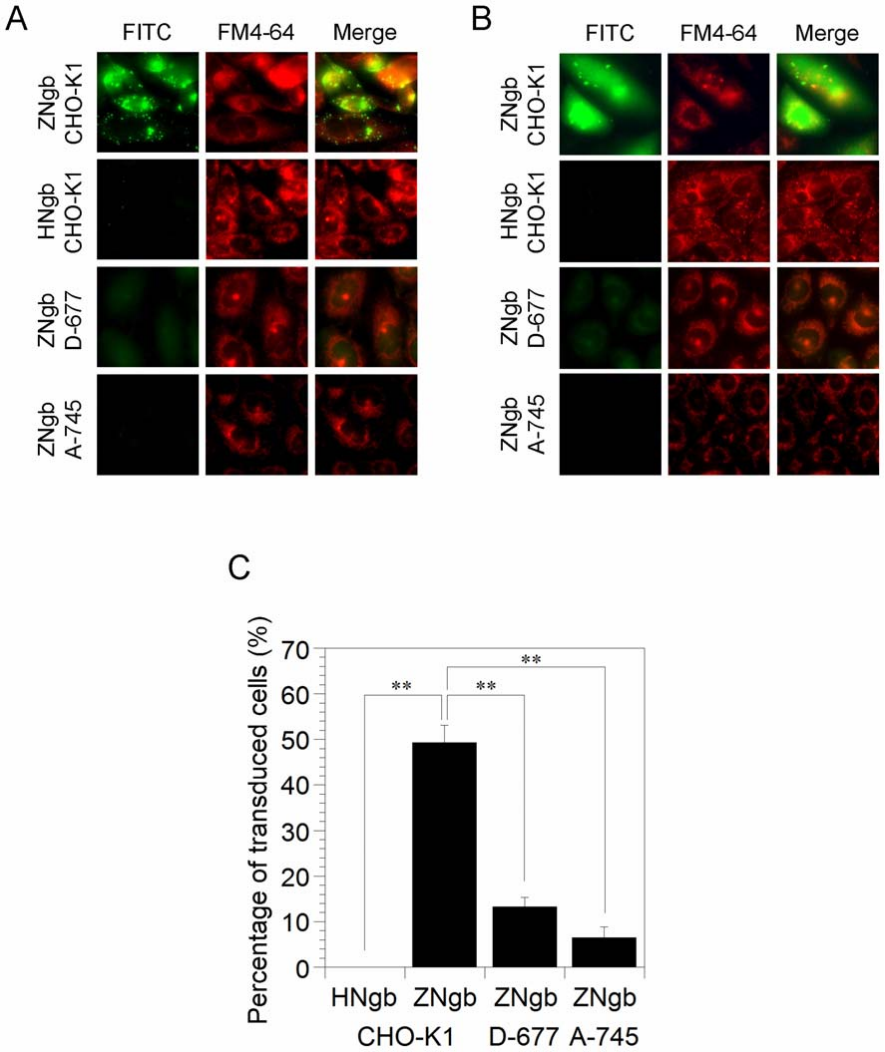
Transduction of zebrafish Ngb (ZNgb) and human Ngb (HNgb) in CHO-K1 and glycosaminoglycan-deficient mutant cell lines. (A,B) Images of FITC-labeled ZNgb and HNgb in CHO-K1 and glycosaminoglycan-deficient mutant cell lines. Each FITC-labeled (green) Ngb protein was applied at 1 µM to cells seeded on glass in the presence of FM4-64 (red). The cells were then incubated for 6 h (A) or 24 h (B) under normoxic conditions. The living, unfixed cells were directly observed by fluorescence microscopy. (C) Percentages of transduced cells showing FITC signal after a 24-h incubation in the fluorescence microscopy assays, from a random selection of fields including at least one hundred cells in total. All data are expressed as means ± standard error of means (SEM) from three independent experiments. ** *P*<0.01, one-way ANOVA.

**Figure 9:**
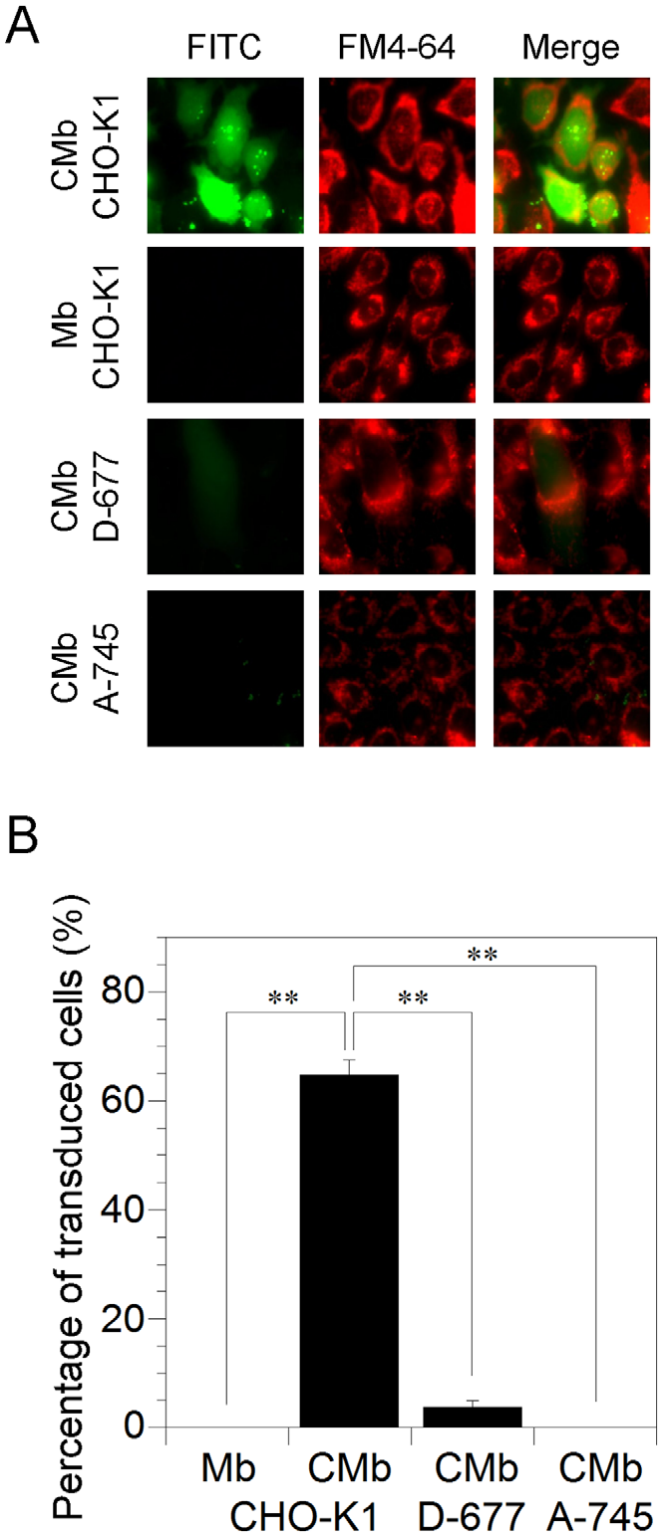
Transduction of human wild-type Mb and chimeric Mb (CMb) in CHO-K1 and glycosaminoglycan-deficient mutant cell lines. (A) Images of FITC-labeled Mb and CMb in CHO-K1 and glycosaminoglycan-deficient mutant cell lines after a 24-h incubation. Each FITC-labeled (green) protein was applied at 1 µM to the cells seeded on glass in the presence of FM4-64 (red). The cells were then incubated for 24 h under normoxic conditions. The living, unfixed cells were directly observed by fluorescence microscopy. (B) Percentages of transduced cells showing FITC signal after a 24-h incubation in the fluorescence microscopy assays, from a random selection of fields including at least one hundred cells in total. All data are expressed as means ± standard error of means (SEM) from three independent experiments. ** *P*<0.01, one-way ANOVA.

To investigate the interaction of Ngb with glycosaminoglycan (GAG), we used a well-characterized wild-type Chinese hamster ovary CHO cell line, CHO-K1, and two mutants deficient in GAG synthesis, D-677 and A-745. Cell line D-677 has a single mutation that affects both N-acetylglucosaminyltransferase and glucuronosyltransferase activities, which are necessary for the polymerization of heparan sulfate disaccharide chains, and thus D-677 cells do not synthesize any heparan sulfate proteoglycans [33]. Moreover, the D-677 cell line produces approximately three times more chondroitin sulfate than wild-type cells [33]. The A-745 cell line lacks xylosyltransferase, an enzyme necessary for the initiation of GAG synthesis, and does not produce detectable levels of any proteoglycans [34]. As shown in Figure 8, zebrafish, but not human, Ngb translocated into CHO-K1 cells. On the other hand, zebrafish Ngb did not penetrate the cell membrane of either of the deficient mutants, D-677 or A-745 (Figure 8), suggesting that cellular uptake of zebrafish Ngb is dependent on cell-surface proteoglycans. Figure 9 shows that chimeric Mb translocated into CHO-K1 cells, but not into the two mutants deficient in GAG synthesis, suggesting that protein transduction of chimeric Mb is a cell-surface proteoglycan-dependent process as is that of zebrafish Ngb.

### Molecular design based on modular structures for the creation of novel functional proteins

In the present study, we successfully engineered a novel cell-membrane-penetrating Mb. Moreover, we previously prepared a chimeric ZHHH Ngb protein, in which module M1 of human Ngb was replaced by that of zebrafish Ngb, and demonstrated that the chimeric Ngb protein can translocate into cells and protect cells against cell death under oxidative stress conditions [17], [21]. These results led us to conclude that module M1 is a structural and functional unit that has the advantage in producing novel stable functional proteins.

Previously, Ngb proteins fused to the basic Arg-rich protein transduction domain of the HIV-1 TAT protein have been reported to translocate into cells efficiently [35]–[37]. However, whereas Zhou et al. reported that rat Ngb fused to TAT could protect cells against apoptosis induced by hypoxia [37], Peroni et al. did not observe protection of cells against the effects of deprivation of oxygen and glucose by exposure to human Ngb fused to TAT [36]. Fusion of Ngb to the TAT sequence might block the binding of Ngb to Gα_i_ and/or induce changes in the Ngb−Gα_i_ interaction, due to the presence of many positively-charged residues within the TAT sequence. Because module M1 of zebrafish Ngb contains less basic residues than the TAT sequence, protein design using module M1 of zebrafish Ngb may have the advantage of retaining natural electrostatic protein-protein interactions.

In addition, we previously fused NH_2_-terminal Rossmann fold modules of human glyceraldehyde-3-phosphate dehydrogenase (GapDH) (amino acids 1-148) to the N-terminus of full-length human Mb to prepare a ‘modules(GapDH)-fused Mb’ protein [38]. We showed that this modules(GapDH)-fused Mb protein binds to heme in a stoichiometric 1∶1 heme:protein ratio as does wild-type Mb, and that the modules(GapDH)-fused Mb protein has a heme environment similar to that of wild-type Mb [38]. Moreover, we demonstrated that the modules(GapDH)-fused Mb protein can bind to the angiostatic form of human tryptophanyl-tRNA synthetase (TrpRS) and stimulate its aminoacylation activity as can wild-type GapDH [38]. It should be also noted that phylogenetically older introns strongly correlate with module boundaries in ancient proteins [39]. These modules of Ngb and GapDH also correspond to exons interrupted by phylogenetically older introns at the DNA level [40]–[43]. From our present results on chimeric Mb, as well as our previous data on several module-substituted proteins [17], [20], [21], [38], [44]–[47], we conclude that module substitutions focused on phylogenetically older introns will be useful for the design and production of novel functional proteins.

## Materials and Methods

### Preparation of wild-type myoglobin (Mb) and chimeric Mb

A cDNA fragment of human Mb was amplified by PCR using human universal Quick-clone cDNA (Clontech, Palo Alto, CA). The gene encoding full-length human Mb, including a COOH-terminal tag of six histidine residues (six-His tag), was cloned into prokaryotic expression vector pET-20b (Novagen, Madison, WI) (Figure 1) [38]. Cys^110^ of human Mb was replaced by Ala to prevent difficulties in protein purification [48]. In this study, we denote this variant of human Mb as “wild-type”. As shown in Figure 1, PCR fragments of zebrafish Ngb module M1 (amino acids 1–30) and human wild-type Mb were cloned into pET-20b to produce a chimeric Mb protein, in which module M1 of zebrafish Ngb (amino acids 1–30) was fused to the NH_2_-terminus of full-length human wild-type Mb with the six-His tag. The constructs were confirmed by DNA sequencing (FASMAC Co., Ltd., DNA sequencing services, Atsugi, Japan). *E. coli* strain BL 21 (DE 3) cells carrying each plasmid were grown in 2xTY culture containing 100 µg/ml of ampicillin and 1.0 mM 5-aminolevulinic acid (Wako Chemicals, Osaka, Japan) at 37C. Overexpression of each Mb protein was induced in BL 21 (DE 3) cells at 37°C after treatment with 0.4 mM isopropyl β-D-thiogalactopyranoside (IPTG) for a further 15 hours. Each protein with the attached six-His tag was purified on a nickel affinity column (His•Bind^®^ resin; Novagen) from the supernatant of lysed cells using the protocol provided by Novagen. Each purified protein was dialyzed overnight against phosphate-buffered saline (PBS). Endotoxin was removed from the protein solutions by phase separation using Triton X-114 (Sigma-Aldrich, St. Louis, MO) [49], [50]. Trace amounts of Triton X-114 were removed by passage through Sephadex G25 gel (GE Healthcare Biosciences, Piscataway, NJ) equilibrated with PBS. The protein concentration was determined spectrophotometrically using an extinction coefficient of 160 mM^−1^cm^−1^ at 409 nm for human ferric Mb [48].

### Preparation of human and zebrafish Ngb

Plasmids for human and zebrafish wild-type Ngb were prepared as described previously [13], [20]. Overexpression of each Ngb protein was induced in *E. coli* strain BL 21 (DE 3) after treatment with IPTG, and each Ngb protein was purified as described previously [13], [17], [20], [21], [27]. In brief, soluble cell extracts were loaded onto DEAE sepharose anion-exchange columns equilibrated with buffer A (20 mM Tris-HCl, pH 8.0). Ngb proteins were eluted from the columns with buffer A containing 75 mM NaCl and further purified by passage through Sephacryl S-200 HR gel filtration columns. Ngb proteins were next applied to a HiTrap Q HP column (GE Healthcare Biosciences), eluted with a 0–500 mM linear NaCl gradient in buffer A. Purified Ngb was dialyzed overnight against PBS. Endotoxin was removed from the protein solutions by phase separation using Triton X-114 (Sigma-Aldrich) [49], [50]. Trace amounts of Triton X-114 were removed by passage through Sephadex G-25 gel (GE Healthcare Biosciences) equilibrated with PBS. The Ngb concentration was determined spectrophotometrically using an extinction coefficient of 122 mM^−1^cm^−1^ at the Soret peak.

### UV-Visible spectra

Electronic absorption spectra of purified proteins were recorded with a UV-visible spectrophotometer (UV-2450; Shimadzu, Kyoto, Japan) at ambient temperature (∼20°C). Spectra were recorded in PBS (pH 7.4).

### Circular dichroism (CD) spectra

CD spectra in the far UV region were measured with a spectropolarimeter (J-805; JASCO Co., Tokyo, Japan) at 20°C. The samples were measured at a concentration of approximately 5 µM in 50 mM sodium phosphate buffer (pH 7.4). The path length of the cells used for the measurements was 1 mm. The molar ellipticity (deg cm^2^ dmol^−1^) was determined on a mean residue basis. The α-helix content (*f*_H_) was calculated according to Chen et al. [51] by the following equation:
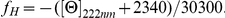



### Denaturation assays

Guanidine hydrochloride (GdnHCl)-induced denaturation experiments were carried out in 50 mM sodium phosphate buffer (pH 7.4), containing various concentrations of GdnHCl. The solutions contained 5 µM protein and were incubated for at least 4 h. CD spectra from 200 to 250 nm were measured. The fractional denatured population (*f*_D_) under each condition was estimated by the following equation:
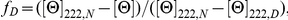
where [*Θ*]_222,N_, [*Θ*]_222,D_, and [*Θ*] represent ellipticities at 222 nm in the native and denatured states, and under each GdnHCl concentration, respectively.

### Gel filtration chromatography

To measure the molecular sizes of proteins, gel filtration chromatography was performed by using a Superdex 200 HR 10/30 column for the FPLC system (GE Healthcare Biosciences). Samples were loaded onto the column equilibrated with 20 mM Tris-HCl, and 150 mM NaCl, pH 8.0, at 4°C.

### Cell culture

HeLa cells (RCB0007) were obtained from the RIKEN Cell Bank. HeLa cells were maintained in culture in Dulbecco's modified Eagle's medium (DMEM) containing 4.5 g/L of glucose, 10% (v/v) fetal bovine serum (FBS), 100 U/ml of penicillin, 100 µg/ml of streptomycin, and 2 mM glutamine (all from Invitrogen) in a humidified atmosphere containing 5% CO_2_ at 37°C. The medium was changed twice weekly, and the cultures were split 1∶8 once every week.

Chinese hamster ovary (CHO) cells (CHO-K1 cell lines: wild-type; GAG-deficient, pgsA-745 (A-745); and heparan sulfate-deficient, pgsD-677 (D-677)) were obtained from the American Type Culture Collection (ATCC; Manassas, VA) and maintained in F-12 nutrient mixture (Ham's F-12) including 2.5 mM glutamine, supplemented with 10% (v/v) FBS, penicillin (100 U/mL), and streptomycin (100 µg/ml). The medium was changed every 3 days, and the cultures were split at a 1∶8 ratio once every week.

### FITC labeling of Ngb and Mb proteins

Ngb or Mb was conjugated to fluorescein isothiocyanate (FITC; Dojindo, Kumamoto, Japan) according to the instructions of a Fluoreporter® FITC protein labeling kit (Molecular Probes, Eugene, OR). FITC-labeled Ngb or Mb was purified using G25 gel chromatography to eliminate free FITC. The concentrations of protein and FITC dye in each purified FITC-labeled protein were calculated on the basis of their absorbance at the Soret peak and 494 nm, respectively. The molar ratio of dye to protein in each purified FITC-labeled protein was determined to be 0.9–1.3 FITC dye molecules per molecule of protein.

### Protein transduction observed by fluorescence microscopy

HeLa cells were seeded at 2×10^4^ cells/ml in 35-mm glass-bottomed dishes (Matsunami Glass, Osaka, Japan). When cells were 60–70% confluent, each FITC-labeled Mb protein was added to cells that had been washed in DMEM without serum, in the presence of 1 µM FM4-64 (Molecular Probes, Eugene, OR), a general fluorescent marker of endocytosis. Fresh DMEM without serum was added and the cells were incubated at 37°C for 1 h; FBS was then added to a final concentration of 2%. The cells were incubated under normoxia at 37°C for the indicated time. HeLa cells were washed with cold PBS twice, and the living, unfixed cells were directly observed by fluorescence microscopy (Olympus IX71, Tokyo, Japan).

CHO-K1, A-745, and D-677 cells were seeded at 1×10^5^ cells/mL in 35-mm glass-bottomed dishes (MatTek Corp., Ashland, MA) and were incubated for 48 h. Each FITC-labeled Ngb or Mb protein was added to cells that had been washed in DMEM without serum, in the presence of 1 µM FM4-64. Fresh DMEM without serum was added and the cells were incubated at 37°C for 1 h; FBS was then added to a final concentration of 2%. The cells were incubated under normoxia at 37°C for the indicated time. Cells were washed with cold PBS twice, and the living, unfixed cells were directly observed by fluorescence microscopy (Olympus IX71).

### Western blot analyses

HeLa cells were seeded at 1×10^5^ cells/ml in 35-mm plastic dishes (Corning Inc, Corning, NY). When cells were 60–70% confluent, chimeric Mb or human wild-type Mb was applied at 5 µM to cells that had been washed in DMEM without serum. Fresh DMEM without serum was added and the cells were incubated at 37°C for 1 h; FBS was then added to a final concentration of 2%. The cells were incubated under normoxia at 37°C for 24 h. After HeLa cells were washed with cold PBS twice, extracts of soluble proteins were prepared. Protein samples were resolved by electrophoresis on 18.0% SDS-polyacrylamide gels. Proteins were electroblotted onto Hybond-P PVDF membranes (GE Healthcare) for 1 h. The membranes were incubated for 1 h with mouse anti-penta-His monoclonal antibody (Qiagen, Valencia, CA) in PBS. After being washed three times with PBS containing 0.1% Tween20, the membranes were incubated with an HRP-linked sheep anti-mouse Ig (GE Healthcare) for 1 h. The membranes were again washed three times with the buffer, and the proteins were visualized using ECL^TM^ western blotting detection reagents (GE Healthcare).

## References

[1] Burmester T, Weich B, Reinhardt S, Hankeln T (2000). A vertebrate globin expressed in the brain.. Nature.

[2] Dewilde S, Kiger L, Burmester T, Hankeln T, Baudin-Creuza V (2001). Biochemical characterization and ligand binding properties of neuroglobin, a novel member of the globin family.. J Biol Chem.

[3] Trent JT, Watts RA, Hargrove MS (2001). Human neuroglobin, a hexacorordinate hemoglobin that reversibly binds oxygen.. J Biol Chem.

[4] Zhang C, Wang C, Deng M, Li L, Wang H (2002). Full-length cDNA cloning of human neuroglobin and tissue expression of rat neuroglobin.. Biochem Biophys Res Commun.

[5] Schmidt M, Gieβl A, Laufs T, Hankeln T, Wolfrum U (2003). How does the eye breathe? Evidence for neuroglobin-mediated oxygen supply in the mammalian retina.. J Biol Chem.

[6] Wystub S, Laufs T, Schmidt M, Burmester T, Maas U (2003). Localization of neuroglobin protein in the mouse brain.. Neurosci Lett.

[7] Sun Y, Jin K, Mao XO, Zhu Y, Greenberg DA (2001). Neuroglobin is up-regulated by and protects neurons from hypoxic-ischemic injury.. Proc Natl Acad Sci USA.

[8] Sun Y, Jin K, Peel A, Mao XO, Xie L (2003). Neuroglobin protects the brain from experimental stroke *in vivo*.. Proc Natl Acad Sci USA.

[9] Fordel E, Thijs L, Martinet W, Lenjou M, Laufs T (2006). Neuroglobin and cytoglobin overexpression protects human SH-SY5Y neuroblastoma cells against oxidative stress-induced cell death.. Neurosci Lett.

[10] Khan AA, Wang Y, Sun Y, Mao XO, Xie L (2006). Neuroglobin-overexpressing transgenic mice are resistant to cerebral and myocardial ischemia.. Proc Natl Acad Sci USA.

[11] Li RC, Pouranfar F, Lee SK, Morris MW, Wang Y (2008). Neuroglobin protects PC12 cells against β-amyloid-induced cell injury.. Neurobiol Aging.

[12] Jin K, Mao Y, Mao X, Xie L, Greenberg DA (2010). Neuroglobin expression in ischemic stroke.. Stroke.

[13] Wakasugi K, Nakano T, Morishima I (2003). Oxidized human neuroglobin as a heterotrimeric Gα protein guanine nucleotide dissociation inhibitor.. J Biol Chem.

[14] Wakasugi K, Kitatsuji C, Morishima I (2005). Possible neuroprotective mechanism of human neuroglobin.. Ann N Y Acad Sci.

[15] Kitatsuji C, Kurogochi M, Nishimura S, Ishimori K, Wakasugi K (2007). Molecular basis of guanine nucleotide dissociation inhibitor activity of human neuroglobin by chemical cross-linking and mass spectrometry.. J Mol Biol.

[16] Morris MC, Depollier J, Mery J, Heitz F, Divita G (2001). A peptide carrier for the delivery of biologically active proteins into mammalian cells.. Nat Biotechnol.

[17] Watanabe S, Wakasugi K (2008). Neuroprotective function of human neuroglobin is correlated with its guanine nucleotide dissociation inhibitor activity.. Biochem Biophys Res Commun.

[18] Awenius C, Hankeln T, Burmester T (2001). Neuroglobins from the zebrafish Danio rerio and the pufferfish Tetraodon nigroviridis.. Biochem Biophys Res Commun.

[19] Fuchs C, Heib V, Kiger L, Haberkamp M, Roesner A (2004). Zebrafish reveals different and conserved features of vertebrate neuroglobin gene structure, expression pattern, and ligand binding.. J Biol Chem.

[20] Wakasugi K, Morishima I (2005). Identification of residues in human neuroglobin crucial for guanine nucleotide dissociation inhibitor activity.. Biochemistry.

[21] Watanabe S, Wakasugi K (2008). Zebrafish neuroglobin is a cell-membrane-penetrating globin.. Biochemistry.

[22] Gilbert W (1978). Why genes in pieces?. Nature.

[23] Blake CC (1979). Exons encode protein functional units.. Nature.

[24] Gō M (1981). Correlation of DNA exonic regions with protein structural units in haemoglobin.. Nature.

[25] Gō M (1983). Modular structural units, exons, and function in chicken lysozyme.. Proc Natl Acad Sci USA.

[26] Gō M (1985). Protein structures and split genes.. Adv Biophys.

[27] Watanabe S, Wakasugi K (2010). Identification of residues critical for the cell-membrane-penetrating activity of zebrafish neuroglobin.. FEBS Lett.

[28] Schwarze SR, Ho A, Vocero-Akbani A, Dowdy SF (1999). In vivo protein transduction: Delivery of a biologically active protein into the mouse.. Science.

[29] Futaki S (2005). Membrane-permeable arginine-rich peptides and the translocation mechanisms.. Adv Drug Deliv Rev.

[30] Noguchi H, Bonner-Weir S, Wei F-Y, Matsushita M, Matsumoto S (2005). BETA2/NeuroD protein can be transduced into cells due to an arginine- and lysine-rich sequence.. Diabetes.

[31] Nakase I, Tadokoro A, Kawabata N, Takeuchi T, Katoh H (2007). Interaction of arginine-rich peptides with membrane-associated protepglycans is crucial for induction of actin organization and macropinocytosis.. Biochemistry.

[32] Noguchi H, Ueda M, Matsumoto S, Kobayashi N, Hayashi S (2007). BETA2/NeuroD protein transduction requires cell surface heparan sulfate proteoglycans.. Hum Gene Ther.

[33] Lidholt K, Weinke JL, Kiser CS, Lugemwa FN, Bame KJ (1992). A single mutation affects both *N*-acetylglucosaminyltransferase and glucuronosyltransferase activities in a Chinese hamster ovary cell mutant defective in heparan sulfate biosynthesis.. Proc Natl Acad Sci USA.

[34] Esko JD, Stewart TE, Taylor WH (1985). Animal cell mutants defective in glycosaminoglycan biosynthesis.. Proc Natl Acad Sci USA.

[35] Mendoza V, Klein D, Ichii H, Ribeiro MM, Ricordi C (2005). Protection of islets in culture by delivery of oxygen binding neuroglobin via protein transduction.. Transplant Proc.

[36] Peroni D, Negro A, Bähr M, Dietz GPH (2007). Intracellular delivery of neuroglobin using HIV-1 TAT protein transduction domain fails to protect against oxygen and glucose deprivation.. Neurosci Lett.

[37] Zhou G-Y, Zhou S-N, Lou Z-Y, Zhu C-S, Zheng X-P (2008). Translocation and neuroprotective properties of TAT PTD Ngb fusion protein in primary cultured cortical neurons.. Biotechnol Appl Biochem.

[38] Wakasugi K, Nakano T, Morishima I (2005). Oxidative stress-responsive intracellular regulation specific for the angiostatic form of human tryptophanyl-tRNA synthetase.. Biochemistry.

[39] Fedorov A, Roy S, Cao X, Gilbert W (2003). Phylogenetically older introns strongly correlate with module boundaries in ancient proteins.. Genome Res.

[40] Shih M-C, Heinrich P, Goodman HM (1988). Intron existence predated the divergence of eukaryotes and prokaryotes.. Science.

[41] Kersanach R, Brinkmann H, Liaud M-F, Zhang D-X, Martin W (1994). Five identical intron positions in ancient duplicated genes of eubacterial origin.. Nature.

[42] Long M, de Souza SJ, Rosenberg C, Gilbert W (1996). Exon shuffling and the origin of the mitochondrial targeting function in plant cytochrome c1 precursor.. Proc Natl Acad Sci USA.

[43] Suzuki T, Imai K (1998). Evolution of myoglobin.. Cell Mol Life Sci.

[44] Wakasugi K, Ishimori K, Imai K, Wada Y, Morishima I (1994). “Module” substitution in hemoglobin subunits. Preparation and characterization of a “chimera βα-subunit”.. J Biol Chem.

[45] Wakasugi K, Ishimori K, Morishima I (1997). ‘Module’-substituted globins: artificial exon shuffling among myoglobin, hemoglobin α- and β-subunits.. Biophys Chem.

[46] Wakasugi K, Quinn CL, Tao N, Schimmel P (1998). Genetic code in evolution:swiching species-specific aminoacylation with a peptide transplant.. EMBO J.

[47] Wakasugi K, Morishima I (2005). Preparation and characterization of a chimeric zebrafish-human neuroglobin engineered by module substitution.. Biochem Biophys Res Commun.

[48] Varadarajan R, Lambright DG, Boxer SG (1989). Electrostatic interactions in wild-type and mutant recombinant human myoglobins.. Biochemistry.

[49] Aida Y, Pabst MJ (1990). Removal of endotoxin from protein solutions by phase separation using Triton X-114.. J Immunol Methods.

[50] Liu S, Tobias R, McClure S, Styba G, Shi Q (1997). Removal of endotoxin from recombinant protein preparations.. Clin Biochem.

[51] Chen YH, Yang JT, Martinez HM (1972). Determination of the secondary structures of proteins by circular dichroism and optical rotatory dispersion.. Biochemistry.

